# A Notable Book on Biological Therapeutics

**Published:** 1914-11

**Authors:** 


A NOTABLE BOOK ON BIOLOGICAL THERAPEUTICS.—A book of uncommon interest and value to physicians has just been issued from the press of Parke, Davis & Co. It is a new "Manual of Biological Therapeutics," receipt of a copy of which is hereby acknowledged by the editor of this JOURNAL. The book is handsomely printed in large, clear type, on heavy enameled paper, and bound in cloth. It contains 174 pages of text, upwards of thirty full-page plates in color, and a number of half-tone illustrations in black and white, together with a comprehensive index. As its title suggests, it is a concise and practical treatise on biological therapeutics, and so replete with useful information that no practitioner should miss the opportunity to secure a copy, especially in view of the fact that the publishers announce that the entire edition is to be distributed gratuitously to members of the medical profession, on individual application.

Something of the scope and value of the work may be inferred from this incomplete list of the subjects treated: Biology; Bacteria; Immunity; The Preparation and Uses of Sera; Antidiphtheric Serum; Concentrated Diphtheria Antitoxin; Allergic Reactions; Antitetanic Serum and Globulins; Antigonococcic Serum; Antimeningitic Serum; Antistreptococcic Serum; Bacterial Vaccines or Bacterins; The Opsonic Index and description of method of taking it; When Serums should be used and when Bacterial Vaccines are to be preferred; The various Bacterins and their Indications; Smallpox Vaccine; Pasteur Antirabic Vaccine; The Diagnosis of Typhoid Fever; The Agglutination Test without a Microscope; The Agglutometer; Ehrlich's Diazo-Reaction in Typhoid Fever; Gonococcus Antigen; The Wassermann Reaction; Coley's Mixture; Coagulose or Hemostatic Ferment; Bacillus Lactis Bulgaricus; Phylacogens, their Preparation and Mode of Use; Mixed Infection Phylacogen; Pneumonia Phylacogen; Gonorrhea Phylacogen; Erysipelas Phylacogen; Rheumatism Phylacogen; Typhoid Phylacogen; Tuberculins in Diagnosis and Treatment; Organotherapy; Thyreoidectin and Thyroprotein; Thyroid and Thymus Glands; Adrenalin and Pituitrin; Corpora Lutea; The Biological Farm and the Research Laboratory.

To our physician friends we suggest the propriety of writing at once for a copy of this "Manual of Biological Therapeutics," addressing the request to Parke, Davis & Co., at their home office in Detroit, Michigan. It will not be amiss to mention this JOURNAL in writing.

